# Renin–angiotensin system inhibitors (RASi) are not nephrotoxic – they protect the kidneys and the heart

**DOI:** 10.1016/j.clinme.2026.100577

**Published:** 2026-03-30

**Authors:** Jonathan S. Murray, Paul R. Kalra, Lisa J. Anderson, Nicholas M. Selby, Simon Sawhney, William S. McKane, Marlies Ostermann, Nitin V. Kolhe, Stephen J McWilliam, Karen Nagalingam, Shelagh Bickerton, Andrew J.P. Lewington, William T. Hinchliffe, Simon J. Smith, Michael Wise, Alan Hancock, Edward Kingdon, Paul Cockwell, Matthew T. James, Thomas Blakeman, John D. Dean, Clare Morlidge, Laurie A. Tomlinson, Darren Green

**Affiliations:** a South Tees Hospitals NHS Foundation Trust, Middlesbrough TS4 3BW, UK; b School of Health & Life Sciences, Centuria Building, Teesside University, Middlesbrough TS1 3BX, UK; c Dept Cardiology, Portsmouth Hospitals University NHS Trust, Cosham PO6 3LY, UK; d Faculty of Science and Health, University of Portsmouth, Portsmouth, UK; e St George's University Hospitals NHS Foundation Trust and City St George’s University of London, UK; f Centre for Kidney Research and Innovation, Academic Unit for Translational Medical Sciences, School of Medicine, University of Nottingham, UK; g Centre for Biostatistics and Health Data Science, Polwarth Building, University of Aberdeen, AB25 2ZD, UK; h Sheffield Teaching Hospitals NHSFT, Sheffield S5 7AU, UK; i King’s College London, Guy’s & St Thomas’ NHS Foundation Trust, Westminster Bridge Road, London SE1 7EH, UK; j Department of Renal Medicine, University Hospitals of Derby and Burton NHS Foundation Trust, Derby, UK; k Division of Medical Sciences and Graduate Entry Medicine, University of Nottingham, Derby, UK; l Department of Women’s and Children’s Health, University of Liverpool, Institute in the Park, Alder Hey Children’s NHS Foundation Trust, Eaton Road, Liverpool L12 2AP, UK; m Lister Hospital, East and North Hertfordshire NHS Trust, Corey Mill Lane, Stevenage SG1 4AJ, UK; n Royal Wolverhampton NHS Trust, WV10 0QP, UK; o St. James's University Hospital, Leeds LS9 7TF, UK; p National Institute for Health and Care Research (NIHR) HealthTech Research Centre, Leeds, UK; q County Durham and Darlington NHS Foundation Trust, University Hospital of North Durham, North Road, Durham DH1 5TW, UK; r Patient Representative; s Sussex Kidney Unit, University Hospitals Sussex NHSFT, Eastern Rd, Brighton, Brighton and Hove BN2 5BE, UK; t Department of Nephrology, Queen Elizabeth Hospital, Mindlesohn Way, Birmingham B15 2WG, UK; u College of Medical and Dental Sciences, University of Birmingham, B15 2TT, UK; v Departments of Medicine and Community Health Sciences, Cumming School of Medicine, University of Calgary, Calgary, Alberta T2N 4Z6, Canada; w Centre for Primary Care and Health Services Research, Division of Population Health, Health Services Research & Primary Care, The University of Manchester, Manchester M13 9PL, UK; x East Lancashire Hospitals NHS Trust, UK; y East and North Herts NHS Teaching Trust, Pharmacy Department, Coreys Mill Lane, Stevenage SG1 4AB, UK; z Faculty of Epidemiology and Population Health, London School of Hygiene and Tropical Medicine, London WC1E 7HT, UK; aa Salford Care Organisation, Northern Care Alliance NHS Foundation Trust, UK; ab University of Manchester, Manchester, UK

**Keywords:** Heart failure, Kidney disease, Medicines safety, Healthcare and patient education

## Abstract

Healthcare professionals should not label medications as ‘kidney toxins’ unless this is actually the case, especially medications that instead confer clear prognostic benefit, such as renin–angiotensin system inhibitors (RASi). This is imperative when discussing treatments with people with long-term health conditions, for whom RASi significantly reduce death, progression of chronic kidney disease (CKD) and hospitalisation. RASi are fundamental to management of heart failure with reduced ejection fraction (HFrEF) and CKD with proteinuria, yet are frequently called ‘nephrotoxic’. The association between RASi use and acute kidney injury (AKI) is too often mistaken for ‘causation’; it is largely driven by the use of RASi to treat long-term conditions that increase AKI risk, such as HFrEF, CKD and diabetes mellitus. Mislabelling RASi as ‘nephrotoxic’ adversely affects vital decision making, driving a tendency for RASi avoidance, even when RASi use has clear prognostic benefit. Healthcare education must embed this clinically important change to convention.

## Graphical Abstract

**Figure:**
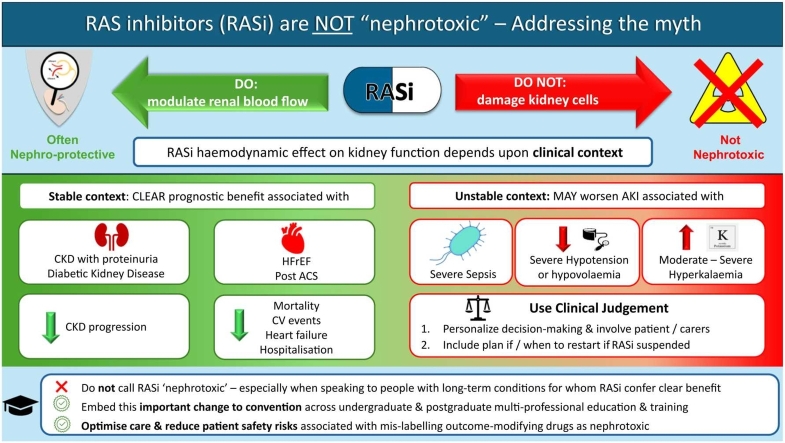

## Background: RASi and other disease-modifying medicines for serious long-term cardiorenal disease

RASi confer significant prognostic benefit for many people living with serious long-term conditions, including HFrEF, CKD and diabetes mellitus.^1^ RASi use is therefore first line within international guidelines^2^, ^3^, ^4^ and these outcome-improving medications are among the most commonly prescribed worldwide.^5^, ^6^ The focus of this article is on RASi, but the principles apply to other medicines used for prognostic advantage in cardiorenal disease, including mineralocorticoid receptor antagonists (MRAs) and sodium glucose co-transporter 2 inhibitors (SGLT2i).

**Fig. 1:**
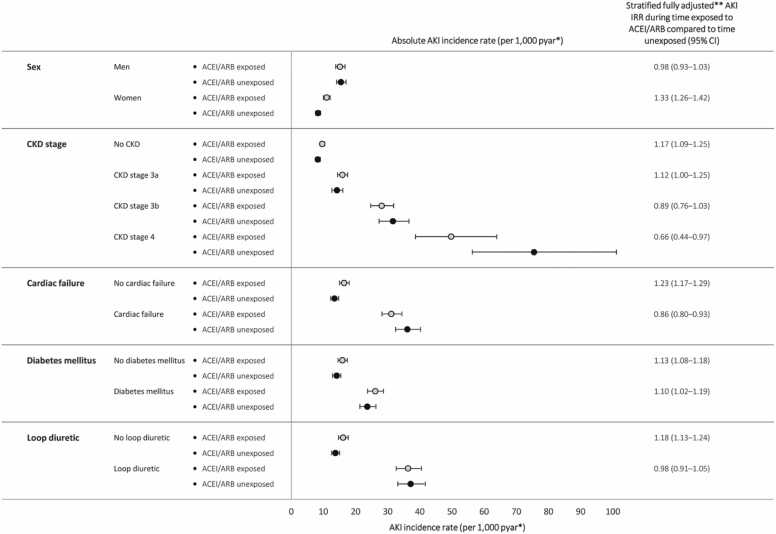
Modelled rates of AKI per 1,000 person years at risk for AKI during time exposed to antihypertensive treatment including angiotensin converting enzyme inhibitors / angiotensin II receptor blockers (ACEI/ARB) compared to time exposed to antihypertensive treatment excluding ACEI/ARB, stratified by characteristics and comorbidities.

Reproduced from Mansfield *et al.,*^8^ distributed under the terms of the Creative Commons Attribution Non-Commercial (CC BY NC 4.0) licence.

Heart failure is the commonest cause of hospitalisation in older people, and a comorbid finding in 14% of inpatients.^7^ People with heart failure are at greater risk of AKI than the general population, especially during acute illness. Large observational analyses suggest that their increased AKI risk is predominantly associated with patient characteristics and comorbidities, with smaller risks associated with RASi therapy.^8^ Moreover, RASi use is *not* associated with substantially greater risk of AKI than use of other antihypertensive medications in people with heart failure (Fig. 1). Similarly, systematic review and meta-analysis suggest that SGLT2i use does not increase incidence of AKI among patients treated for heart failure with these disease-modifying medications.^9^

**Fig. 2:**
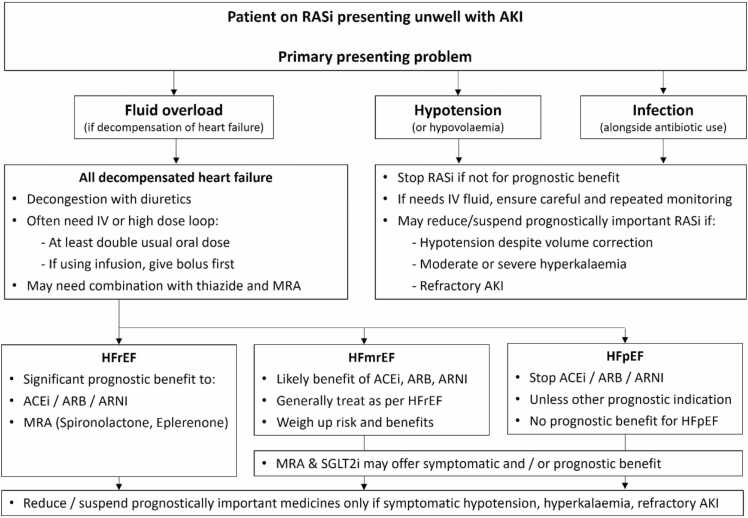
Management of patients with AKI or worsening renal function who are receiving RASi. IV, intravenous; ACEi, angiotensin converting enzyme inhibitors; ARB, angiotensin receptor blockers; MRA, mineralocorticoid receptor antagonist; ARNI, angiotensin receptor/neprilysin inhibitor; SGLT2i, Sodium Glucose Co-transporter 2 inhibitors; HFrEF, heart failure with reduced ejection fraction; HFmrEF, heart failure with mildly reduced ejection fraction; HFpEF, heart failure with preserved ejection fraction.

Adapted from Clark *et al.*,^11^ distributed under the terms of the Creative Commons Attribution Non-Commercial (CC BY NC 4.0) licence.

When people with HFrEF sustain AKI, balancing the need to maintain kidney perfusion while achieving fluid loss requires timely and regular assessment by senior decision makers. It is often assumed that RASi withdrawal is required to achieve both goals, but RASi continuation or dose reduction may be preferable to discontinuation, even in the face of AKI.^10^ Exceptions to this include severe hypotension or moderate to severe hyperkalaemia^11^ (Fig. 2).

RASi exert renal haemodynamic effects that reduce intraglomerular pressure and thereby slow progressive nephron loss associated with glomerular hyperfiltration during CKD. This underpins why RASi are a cornerstone therapy for CKD and should generally be considered nephro-*protective* rather than nephro-*toxic*, a concept that is supported by several large-scale studies.

RASi have been shown to significantly slow progression of mild to moderate CKD, especially diabetic kidney disease, an effect that is independent of blood pressure lowering.^12^, ^13^ Meta-analysis suggests that starting RASi therapy in people with more advanced CKD is similarly associated with reduced rates of CKD progression,^14^ while the *STOP-ACEi* trial found that withdrawing RASi did not slow CKD progression in people with stage 4 or 5 CKD.^15^ Moreover, in a large Swedish emulation of STOP-ACE, stopping RASi in patients with advanced CKD was associated with higher absolute risks of mortality and major adverse cardiovascular events, but with lower absolute risk of initiating dialysis.^16^

RASi are also widely used in children with heart failure and/or CKD. Much paediatric RASi therapy is ‘off-label’ and extrapolated from adult practice; the little paediatric-specific evidence available does not suggest that RASi are ‘nephrotoxic’ for children.^17^, ^18^

## Medications that can affect kidney function: nephrotoxins and renal blood flow modulators

Nephrotoxicity is a term conventionally used to describe *structural kidney damage* that occurs following use of various medications.^19^

Truly nephrotoxic medications or substances cause structural kidney damage or inflammation, *regardless of clinical context*. Nephrotoxin-induced kidney dysfunction (reduced glomerular filtration rate, GFR) can thereby occur in people who are otherwise well and may be irreversible, even after drug discontinuation. Examples include aminoglycoside antibiotics and some chemotherapeutics.

In contrast to nephrotoxins, RASi modulate renal blood flow, reducing efferent arteriolar tone and intraglomerular perfusion pressure, but do not cause structural kidney damage or inflammation. The degree and clinical implications of RASi-associated haemodynamic changes upon kidney function (GFR) may be *affected by clinical context*.

A modest and relatively stable GFR drop is commonly observed after RASi initiation or dose increase in *stable clinical circumstances*. This GFR change is usually predictable and acceptable, as it typically reflects a reversible and nephro-protective reduction in intraglomerular perfusion pressure. A *very rare exception* can arise in people with haemodynamically significant renovascular disease; RASi may cause more marked and *rapidly progressive* reduction of GFR in this context.

RASi-associated haemodynamic changes can affect autoregulation of renal blood flow, which may be required to maintain adequate intraglomerular perfusion pressure, during some *unstable clinical circumstances*. This concept underpins why AKI associated with severe hypotension, hypovolaemia or sepsis may be exacerbated by RASi and, if suspended, RASi can usually be restarted promptly after acute illness recovery. Moreover, incomplete recovery of renal function should not preclude timely reinitiation of RASi therapy, especially when otherwise clinically indicated.^20^

## Patient safety risks associated with referring to RASi as nephrotoxins

Mislabelling RASi medication as ‘nephrotoxic’ risks patients and healthcare professionals misunderstanding the overall clinical ‘benefit over risk’ profile of these medications. This can negatively influence clinical decision making, therapeutic trust and practice; healthcare professionals and patients will be understandably reluctant to use medications considered ‘toxic to their kidneys’.

Despite clear evidence and recommendations supporting RASi use, RASi are frequently avoided, or suspended and not restarted within a clinically effective timeframe. In a large Canadian cohort, post-discharge RASi use was associated with lower mortality among patients hospitalised with AKI.^21^ However, discharge communication following AKI in hospital is often inadequate or untimely^22^ and in England only 60% of patients hospitalised with AKI had RASi restarted within 90 days of discharge.^23^ This risks destabilisation of common long-term health conditions^24^ and may thereby underlie many serious adverse patient outcomes that often occur post AKI, including high mortality and emergency hospital readmission, often due to acute pulmonary oedema.^25^ Considerations to support safe and timely RASi reintroduction in such circumstances are summarised in Table 1.

**Table 1:** Considerations to support safe and timely reintroduction of RASi if suspended during AKI (adapted from 10, 26, 27).

Consideration	Key points
1. General principles	• Use overall clinical judgement and individualise decisions.
	• Many patients receiving RASi are at ongoing high AKI risk because of their comorbidities, but also gain major prognostic benefit from RASi.
	• Restart should be prioritised for HFrEF, CKD and diabetes to prevent destabilising these conditions, as soon as clinically appropriate, and not delayed by incomplete renal recovery.
2. Holistic and shared decisions	• Wherever possible, stopping or restarting should be agreed with the patient, documented, and communicated with a clear follow-up plan (who reviews, where and when, ideally within 2 weeks for high-risk patients^28^).
	• Omitting restart without discussion or documentation should be avoided.
3. Indications for RASi	• Strongly prioritise restart when there is a compelling indication: HFrEF, proteinuric CKD or diabetes.
4. Clinical status	• Restart once the acute illness/instability has resolved.
	• Assess fluid status; in congestion (especially HFrEF), consider optimising diuretics alongside/before RASi.
5. Blood pressure	• Restart at a low threshold if blood pressure is elevated or at the patient’s usual level.
	• Low blood pressure may be the patient’s baseline, and RASi benefit in HFrEF/CKD is often independent of blood pressure lowering.
6. Kidney function	• Do not delay restart solely because renal function has not fully returned to baseline. Interpret current kidney function in the context of baseline and prior trajectory.
	• Consider renal advice if there is new or rapidly progressive CKD after acute illness recovery, particularly with proteinuria, as per CKD guidelines^29^
7. Serum potassium	• Review recent potassium values. If previously high, address modifiable factors (diet, interacting drugs, potassium binders) and arrange early repeat blood tests.
8. Other drugs	• If other disease-modifying agents were held during AKI (eg MRAs, SGLT2 inhibitors), consider phased reintroduction.
	• Aim for at least low doses of all indicated classes (eg in HFrEF), balancing blood pressure and potassium.
	• SGLT2 inhibitors have minimal blood pressure effect and may help limit potassium rise, supporting reintroduction of RASi and/or MRAs.

RASi, renin-angiotensin system inhibitor; AKI, acute kidney injury; CKD, chronic kidney disease; HFrEF, heart failure with reduced ejection fraction; MRA, mineralocorticoid receptor antagonist; SGLT2i, Sodium Glucose Co-transporter 2 inhibitor.

## RASi and sick day advice

Generic sick day advice (SDA) incorporating medication changes aims to limit patient harm during acute illness episodes.^30^, ^31^ While *disease-specific* SDA, for example in Addison’s disease or type I diabetes, can be critical, evaluation suggests that SDA understanding and application is less clear when aiming to prevent AKI and its complications.^32^, ^33^, ^34^ Furthermore, there is little evidence that *RASi-specific* SDA prevents AKI,^35^, ^36^ even for people with CKD.^37^

Inappropriate suspension of medications may further risk patient safety and increase overall healthcare service use. For example, the Stopping Perioperative ACE-inhibitors or angiotensin-II receptor blockers (SPACE) trial found that suspending RASi before non-cardiac surgery may be associated with increased risk of clinically significant acute hypertensive episodes, without reducing AKI rates,^38^ while the much larger Stop-or-Not Trial in cardiac surgery showed that stopping RASi made no difference to outcomes or AKI rates.^39^

In summary, while it may be appropriate to suspend or reduce RASi during AKI associated with severe hypotension, hypovolaemia, sepsis or moderate to severe hyperkalaemia, there is little evidence to show that routine adoption of SDA for this purpose is of overall benefit. Furthermore, SDA may risk overall patient harm if applied indiscriminately in heterogeneous and complex clinical scenarios associated with AKI, especially if SDA does not include if/when to restart RASi and/or implies that these medications are nephrotoxic.

## Discussion

Healthcare professionals should avoid misinforming patients or colleagues that RASi are ‘nephrotoxic’, as this risks patient harm due to suboptimal treatment of common and serious long-term conditions.

Unlike truly nephrotoxic medications, RASi do not cause structural kidney damage. Changes to kidney function associated with RASi reflect changes to renal blood flow that are usually nephroprotective. This fundamental concept is supported by large studies showing that RASi slow progression of CKD, underlining that a creatinine rise associated with RASi use is generally safe and beneficial, as it reflects a protective effect upon renal blood flow for most patients.

While it may be appropriate to reduce or suspend RASi during acute illness in the context of a clear and objective indication, such as severe hypotension or hyperkalaemia, timely RASi reinitiation following recovery is often crucial, and if otherwise indicated, should not be delayed due to incomplete renal recovery. Timely individualised communication between patients and healthcare professionals is essential to ensure this.

Undergraduate and postgraduate multiprofessional training must embed these significant changes to convention, to optimise contemporary clinical practice and reduce patient safety risks.

## Take-home messages

1. Healthcare professionals must avoid informing patients that medications are ‘toxic to their kidneys’ unless this is actually the case. Mislabelling RASi as ‘nephrotoxic’ seriously risks patient safety by (a) driving a tendency for these disease-modifying medications to be avoided and (b) the correct cause and treatment of AKI episodes being delayed or missed if AKI episodes are inappropriately attributed to RASi use.
2. The term ‘nephrotoxin’ should be reserved for medications that commonly cause structural injury or inflammation to nephrons, regardless of clinical context. True nephrotoxins can cause structural kidney damage and dysfunction in people who are otherwise well. RASi do not exert any toxic effect upon nephrons, and changes to kidney function associated with RASi use reflect modulation of renal blood flow that is usually nephroprotective.
3. Observational data and large-scale studies show no significant increase in AKI among people treated with RASi compared to those treated with other antihypertensive medications, including people with common and serious long-term health conditions, for whom RASi therapy confers significant prognostic benefits.
4. While it may be appropriate to suspend or reduce RASi dose during AKI episodes associated with severe hypotension, hypovolaemia or moderate to severe hyperkalaemia (Fig. 2), there is little evidence to support routine use of RASi sick day advice to prevent AKI, and some evidence to suggest that stopping RASi inappropriately may cause patient harm.
5. If suspended during AKI, a plan should be documented to restart RASi and other guideline-indicated medications as soon as clinically appropriate, to avoid decompensation of serious long-term conditions. This requires clear and timely communication between healthcare professional teams and patients, including at the point of hospital discharge. Incomplete recovery of renal function post-AKI should not preclude timely reintroduction of RASi, unless the patient remains at high risk of recurrent AKI due to volume depletion, in the context of a high-output stoma, for example.^40^

## CRediT authorship contribution statement

**Alan Hancock:** Writing – review & editing, Conceptualization. **Simon Sawhney:** Writing – review & editing, Writing – original draft, Data curation, Conceptualization. **Edward Kingdon:** Writing – review & editing, Writing – original draft, Conceptualization. **William S. McKane:** Writing – review & editing, Writing – original draft, Data curation, Conceptualization. **Michael Wise:** Writing – review & editing, Conceptualization. **Nicholas M. Selby:** Writing – review & editing, Writing – original draft, Data curation, Conceptualization. **Thomas Blakeman:** Writing – review & editing, Writing – original draft, Conceptualization. **Stephen J. McWilliam:** Writing – review & editing, Writing – original draft, Data curation. **John D. Dean:** Writing – review & editing, Writing – original draft, Conceptualization. **Karen Nagalingam:** Writing – review & editing, Writing – original draft, Data curation. **Paul Cockwell:** Writing – review & editing, Writing – original draft, Conceptualization. **Marlies Ostermann:** Writing – review & editing, Writing – original draft, Data curation, Conceptualization. **Matthew T. James:** Writing – review & editing. **Nitin V. Kohle:** Writing – review & editing, Writing – original draft, Data curation, Conceptualization. **Darren Green:** Writing – review & editing, Writing – original draft, Visualization, Project administration, Data curation, Conceptualization. **William T. Hinchliffe:** Writing – review & editing. **Paul R. Kalra:** Writing – review & editing, Writing – original draft, Data curation, Conceptualization. **Simon J. Smith:** Writing – review & editing, Visualization. **Lisa J. Anderson:** Writing – review & editing, Writing – original draft, Visualization, Data curation, Conceptualization. **Claire Morlidge:** Writing – review & editing, Writing – original draft, Project administration, Data curation, Conceptualization. **Shelagh Bickerton:** Writing – review & editing, Writing – original draft, Data curation, Conceptualization. **Laurie A. Tomlinson:** Writing – review & editing, Writing – original draft, Visualization, Data curation, Conceptualization. **Andrew J.P. Lewington:** Writing – review & editing, Writing – original draft, Conceptualization. **Jonathan S. Murray:** Writing – review & editing, Writing – original draft, Visualization, Project administration, Data curation, Conceptualization.

## Funding

This research did not receive any specific grant from funding agencies in the public, commercial or not-for-profit sectors.

## Declaration of competing interest

JSM is co-chair for the UK Kidney Association (UKKA) Acute Kidney Injury Specialist Interest Group and an expert nephrology adviser for the *British Medical Journal* (both unpaid roles). His contribution to this article is in a personal capacity and the content of the article does not necessarily reflect the position of the UKKA or *British Medical Journal*.

DG reports a relationship with AstraZeneca UK Limited that includes: consulting or advisory and speaking and lecture fees. DG reports a relationship with Boehringer Ingelheim Ltd that includes: consulting or advisory and speaking and lecture fees. DG reports a relationship with Bayer plc that includes: consulting or advisory and speaking and lecture fees. He reports a relationship with Novartis Pharmaceuticals UK that includes: speaking and lecture fees. His contribution to this article is in a personal capacity and the content of the article does not necessarily reflect the position of these roles.

LJA reports a relationship with the British Society for Heart Failure that includes: board membership. Her contribution to this article is in a personal capacity and the content of the article does not necessarily reflect the position of the British Society for Heart Failure.

PRK reports a relationship with FIRE-1 that includes: funding grants. PRK reports a relationship with Pharmacosmos AS that includes: consulting or advisory, funding grants, and speaking and lecture fees. PRK reports a relationship with AstraZeneca that includes: consulting or advisory and speaking and lecture fees. PRK reports a relationship with Boehringer Ingelheim GmbH that includes: consulting or advisory. PRK reports a relationship with Bayer AG that includes: speaking and lecture fees. PRK reports a relationship with Vifor Pharma Switzerland SA that includes: consulting or advisory and speaking and lecture fees. PRK reports a relationship with Novartis that includes: speaking and lecture fees.

SB declares that she is current National Acute Kidney Injury Community of Practice Lead for the Association of Nephrology Nurses UK (unpaid). Her contribution to this article is in a personal capacity and the content of the article does not necessarily reflect the position of the Association of Nephrology Nurses UK.

PC reports the following advisory or leadership roles: past president and trustee UK Kidney Association; other interests or relationships: Boehringer Ingelheim – non-remunerated research collaboration, AstraZeneca – clinical development programme and advisory board, Vifor, advisory board.

TB declares no financial competing interests, though declares the following (non-funded) positions: NHS England Think Kidneys Programme Board Member (2014–17); Royal College of General Practitioners’ AKI Clinical Champion (2017–20); NHSE Renal Services Transformation Programme Post‑AKI care Lead (2021–23); Specialist Committee Member for NICE AKI Quality Standard (QS76) (2022–23); Kidney Disease Improving Global Outcomes (KDIGO) AKI Guideline Work Group (2023‑To date). His contribution to this article is in a personal capacity and the content of the article does not necessarily reflect the position of these roles.

CM is a consultant renal pharmacist, co-chair for the UK Kidney Association (UKKA) Acute Kidney Injury Specialist Interest Group and president of the UKKA. Her contribution to this article is in a personal capacity and the content of the article does not necessarily reflect the position of the UKKA.

MW, AH, WTH, NMS, WSM, SS, KN, LAT, NVK, MO, SJM, MTJ, JDD, AJPL and SJS declare no conflicts of interest.

## References

[1] Jiménez-Marrero S., Cainzos-Achirica M., Monterde D. (2024). Serum potassium abnormalities, renin-angiotensin-aldosterone system inhibitor discontinuation, and clinical outcomes in patients with chronic cardiovascular, metabolic, and renal conditions: a population-based analysis. Eur J Intern Med.

[2] Recommendations. Chronic kidney disease: assessment and management. Guidance | NICE 2021; 2026.

[3] KDIGO 2024 clinical practice guideline for the evaluation and management of chronic kidney disease. 10.1016/j.kint.2023.10.018 38490803

[4] Mcdonagh T.A., Metra M., Adamo M. (2025). 2023 Focused update of the 2021 ESC guidelines for the diagnosis and treatment of acute and chronic heart failure. Eu Heart J.

[5] NHS Business Services Authority. Prescription cost analysis England 2022/23; 2023.

[6] Fuentes A.V., Pineda M.D., Venkata K. (2018). Comprehension of top 200 prescribed drugs in the us as a resource for pharmacy teaching, training and practice. Pharmacy.

[7] Owens P.L., Liang L., Barrett M.L., Fingar K.R. (2006). Anonymous Healthcare Cost and Utilization Project (HCUP) Statistical Briefs.

[8] Mansfield K.E., Nitsch D., Smeeth L., Bhaskaran K., Tomlinson L.A. (2016). Prescription of renin–angiotensin system blockers and risk of acute kidney injury: a population-based cohort study. BMJ Open.

[9] Wang X., He M., Jin D., Sun C., Lu H. (2024). Effect of SGLT-2 inhibitors on acute kidney injury in patients with heart failure: a systematic review and meta-analysis. Diabetol Metab Syndr.

[10] Parikh C.R., Coca S.G. (2019). Permissive AKI” with treatment of heart failure. Kidney Int.

[11] Clark A.L., Kalra P.R., Petrie M.C. (2019). Change in renal function associated with drug treatment in heart failure: national guidance. Heart.

[12] Lewis E.J., Hunsicker L.G., Clarke W.R. (2001). Renoprotective effect of the angiotensin-receptor antagonist irbesartan in patients with nephropathy due to type 2 diabetes. N Engl J Med.

[13] Brenner B.M., Cooper M.E., De Zeeuw D. (2001). Effects of losartan on renal and cardiovascular outcomes in patients with type 2 diabetes and nephropathy. N Engl J Med.

[14] Ku E., Inker L.A., Tighiouart H. (2024). Angiotensin-converting enzyme inhibitors or angiotensin-receptor blockers for advanced chronic kidney disease: a systematic review and retrospective individual participant-level meta-analysis of clinical trials. Ann Intern Med.

[15] Bhandari S., Mehta S., Khwaja A. (2022). Renin–angiotensin system inhibition in advanced chronic kidney disease. N Engl J Med.

[16] Fu E.L., Evans M., Clase C.M. (2021). Stopping renin-angiotensin system inhibitors in patients with advanced CKD and risk of adverse outcomes: a nationwide study. J Am Soc Nephrol.

[17] Terano C., Ishikura K., Miura M. (2016). Incidence of and risk factors for severe acute kidney injury in children with heart failure treated with renin–angiotensin system inhibitors. Eur J Pediatr.

[18] Hirano D., Miwa S., Kakegawa D. (2021). Impact of acute kidney injury in patients prescribed angiotensin-converting enzyme inhibitors over the first two years of life. Pediatr Nephrol.

[19] Džidić-Krivić A., Sher E.K., Kusturica J. (2024). Unveiling drug induced nephrotoxicity using novel biomarkers and cutting-edge preventive strategies. Chem-Biol Interact.

[20] Frances C.D., Noguchi H., Massie B.M., Browner W.S., McClellan M. (2000). Are we inhibited?: Renal insufficiency should not preclude the use of ACE inhibitors for patients with myocardial infarction and depressed left ventricular function. Arch Intern Med.

[21] Brar S., Ye F., James M.T. (2018). Association of angiotensin-converting enzyme inhibitor or angiotensin receptor blocker use with outcomes after acute kidney injury. JAMA Intern Med.

[22] Choon X.Y., Lumlertgul N., Cameron L. (2021). Discharge documentation and follow-up of critically ill patients with acute kidney injury treated with kidney replacement therapy: a retrospective cohort study. Front Med.

[23] Bidulka P., Fu E.L., Leyrat C. (2020). Stopping renin-angiotensin system blockers after acute kidney injury and risk of adverse outcomes: parallel population-based cohort studies in English and Swedish routine care. BMC Med.

[24] Janse R.J., Fu E.L., Clase C.M. (2022). Stopping versus continuing renin–angiotensin–system inhibitors after acute kidney injury and adverse clinical outcomes: an observational study from routine care data. Clin Kidney J.

[25] Sawhney S., Marks A., Fluck N. (2017). Acute kidney injury as an independent risk factor for unplanned 90-day hospital readmissions. BMC Nephrol.

[26] When or if to re-start ACEI, ARB, diuretics and other antihypertensive drugs after an episode of acute kidney injury; 2016.

[27] UK Kidney Association. UKKA AKI summit report + recommendations. London: UK Kidney Association; 2024. Available from: 〈https://www.ukkidney.org/sites/default/files/documents/UKKA%20AKI%20Summit%20Report%20%2B%20Recommendations_compressed.pdf〉.

[28] Tsang J.Y., Murray J.Y., Kingdon E. (2020). Guidance for post-discharge care following acute kidney injury: an appropriateness ratings evaluation. BJGP Open.

[29] Recommendations. Chronic kidney disease: assessment and management. Guidance | NICE 2021; 2026.

[30] Down S. (2020). How to advise on sick day rules. Diabetes Prim Care.

[31] Watson K.E., Dhaliwal K., Robertshaw S. (2023). Consensus recommendations for sick day medication guidance for people with diabetes, kidney, or cardiovascular disease: a modified Delphi process. Am J Kidney Dis.

[32] Morris R.L., Ashcroft D., Phipps D. (2016). Preventing acute kidney injury: a qualitative study exploring ‘sick day rules’ implementation in primary care. BMC Fam Pract.

[33] Martindale A., Elvey R., Howard S.J. (2017). Understanding the implementation of ‘sick day guidance’ to prevent acute kidney injury across a primary care setting in England: a qualitative evaluation. BMJ Open.

[34] Doerfler R.M., Diamantidis C.J., Wagner L. (2019). Usability testing of a sick-day protocol in CKD. Clin J Am Soc Nephrol.

[35] Tomson C., Tomlinson L.A. (2019). Stopping RAS inhibitors to minimize AKI. Clin J Am Soc Nephrol.

[36] Watson K.E., Dhaliwal K., Mcmurtry E. (2022). Sick day medication guidance for people with diabetes, kidney disease, or cardiovascular disease: a systematic scoping review. Kidney Med.

[37] Fink J.C., Maguire R.M., Blakeman T. (2022). Medication holds in CKD during acute volume-depleting illnesses: a randomized controlled trial of a “sick-day” protocol. Kidney Med.

[38] Ackland G.L., Patel A., Abbott T.E.F. (2023). Discontinuation vs. continuation of renin-angiotensin system inhibition before non-cardiac surgery: the SPACE trial. Eur Heart J.

[39] Legrand M., Falcone J., Cholley B. (2024). Continuation vs discontinuation of renin-angiotensin system inhibitors before major noncardiac surgery: the stop-or-not randomized clinical trial. JAMA.

[40] Bickerton S., Catchpole L., Harris K. (2025). Reducing the incidence and severity of acute kidney injury in people with ileostomies—a nurse-led trifecta approach in a district general hospital in the United Kingdom. Nephrol Dial Transpl.

